# Alpha-D-glucan-based vaccine adjuvants: Current status and future perspectives

**DOI:** 10.3389/fimmu.2022.858321

**Published:** 2022-08-31

**Authors:** Veerupaxagouda Patil, Juan F. Hernandez-Franco, Harm HogenEsch, Gourapura J. Renukaradhya

**Affiliations:** ^1^ Center for Food Animal Health, Ohio Agricultural Research and Development Center, Wooster, OH, United States; ^2^ Department of Animal Sciences, The Ohio State University, Columbus, OH, United States; ^3^ Department of Comparative Pathobiology, College of Veterinary Medicine, Purdue University, West Lafayette, IN, United States

**Keywords:** phytoglycogen, nanovaccine, mucosal immune system in pigs, intranasal delivery, pigs

## Abstract

Nanoparticles (NPs) are increasingly used as efficient vaccine antigen-delivery platforms and vaccine adjuvants. Alpha (α)-D-glucans are polysaccharide polymers found in plants, animals, and microbes. Phytoglycogen (PG) is a densely branched dendrimer-like α-D-glucan that forms nanoparticle structures. Two simple chemical modifications of corn-derived PG create positively charged, amphiphilic nanoparticles, known as Nano-11, that stimulate immune responses when used as vaccine adjuvant in a variety of species. Nano-11 is a versatile adjuvant that can be used for alternative routes of vaccination and in combination with other immunostimulatory molecules. This review discusses our current understanding of the mechanism of action of Nano-11 and its future potential applications in animal vaccines.

## Introduction

Nanoparticles (NPs) of plant origin are of interest for pharmaceutical and biomedical applications because of their cost-effectiveness, biodegradability, biocompatibility, stability, and safety. Alpha-D-glucans are ubiquitous natural polysaccharide polymers found in plants, animals, and microbes that have limited bioactivity due to their hydrophobic nature. Several derivatization strategies have been developed to augment both bioavailability and bioactivity. Nano-11 are NPs derived from phytoglycogen (PG); a highly branched form of α-D-glucan obtained from sweet corn. Chemical treatment results in positively charged, amphiphilic NPs with immunostimulatory properties.

## Nanoparticles and their significance

The need for improved vaccine technologies is exemplified by the raging pandemic coronavirus disease 2019 (COVID-19) and the 2009 H1N1 swine influenza pandemic (1). Furthermore, diseases caused by existing and emerging viruses such as porcine epidemic diarrhea virus, influenza and African swine fever, place a significant economic burden on the global swine industry posing threats to food security and public health.

Traditional approaches such as intramuscular administration of inactivated and modified live virus vaccines are plagued by inadequate immunity and safety under field conditions. Therefore, novel advancements are required to augment protection against evolving virus strains and subtypes and to distinguish between vaccinated and infected animals. Nanoparticle-based vaccine formulations offer significant potential for novel vaccination strategies by virtue of their unique physicochemical properties. Their size facilitates uptake by dendritic cells (DCs) and allows administration *via* alternative routes such as intranasal, oral, or intradermal routes which can augment the immune response by targeting tissues rich in antigen-presenting cells and avoids the use of needles. The term “nanoparticles” refers to non-replicative, nanoscale structures generated by recombinant expression or chemical reactions (2). Although the use of NPs in vaccines has tremendous potential, the adoption of synthetic NPs has been slowed by difficulties in scaling-up production, lack of stability and high cost. Plant-derived NPs may be able to overcome these challenges, in particular for animal vaccines.

## Physical characteristics of Nano-11

Nano-11 is derived from a genetic variety of sweet corn (*sugary-1*) that lacks a starch debranching enzyme needed to produce starch (3). In the absence of this enzyme, the kernels form NPs comprised of highly branched polymers of glucose molecules, PG. The abundance of hydroxyl groups on the surface of PG NPs allows for functionalization through chemical modifications. A two-step chemical modification grafts the surface with octenylsuccinate (OS) and (3-chloro-2-hydroxypropyl)-trimethylammonium chloride (CHPTAC), resulting in Nano-11 (4). OS endows the NPs with negative charge and hydrophobicity (5, 6), while an excess of CHPTAC gives Nano-11 an overall positive surface charge over a wide pH range. The NPs are approximately 70-80 nm in diameter with an irregular, cauliflower-like surface (4). Homogenization of PG nanoparticles with a mixture of glycidyltrimethylammonium chloride and sodium hydroxide in water at 45°C for 4 hours, followed by neutralization and precipitation with 95% ethanol also confers positive charge to PG nanoparticles. However, these cationic nanoparticles lack intrinsic adjuvanticity (7). Nano-11 is readily dispersed in aqueous solutions and is highly stable as it can be lyophilized and autoclaved. Nano-11 has a high adsorptive capacity for negatively charged molecules such as proteins and nucleic acids and is a suitable vehicle to deliver other immunostimulants such as the toll-like receptor-3 (TLR-3) agonist polyriboinosinic:polyribocytidylic acid [poly(I:C)] and cyclic dinucleotides which activate the intracellular stimulator of interferon genes (STING) receptor (8, 9). Such combination adjuvants have the potential to be tailored to induce the desired magnitude and type of immune response. Initial studies in mice confirmed that these Nano-11 nanoparticles are safe and efficacious in induction of robust antigen-specific immune responses (4, 10, 11). Subsequently, we also corroborate these findings in pigs vaccinated with Nano-11 adsorbed inactivated swine influenza virus antigen administered intranasally (12, 13). Furthermore, new studies in rainbow trout also proved that PG-derived cationic nanopartcles are safe and efficacious delivery platforms (7, 14). Initial *in vitro* characterization of antigen adsorbed Nano-11 nanoparticles revealed that the protein release pattern during the first 24 hours was ranging from 3-10% depending on the Nano-11 to inactivated swine influenza A whole virus antigen ratios. Furthermore, the overall cumulative release of antigen proteins from Nano-11 particles after 15 days was ranging from 16-26% (12). These results suggest that the adsorbed antigens are quite stable. In our experimental model system, the cationic Nano-11 particles are electrostatically adsorbed with anionic inactivated swine influenza A virus antigen, and this interaction is not interfering with the biological activity *in vitro* and *in vivo* (12, 13).

## Mechanism of action of Nano-11

**Figure 1:**
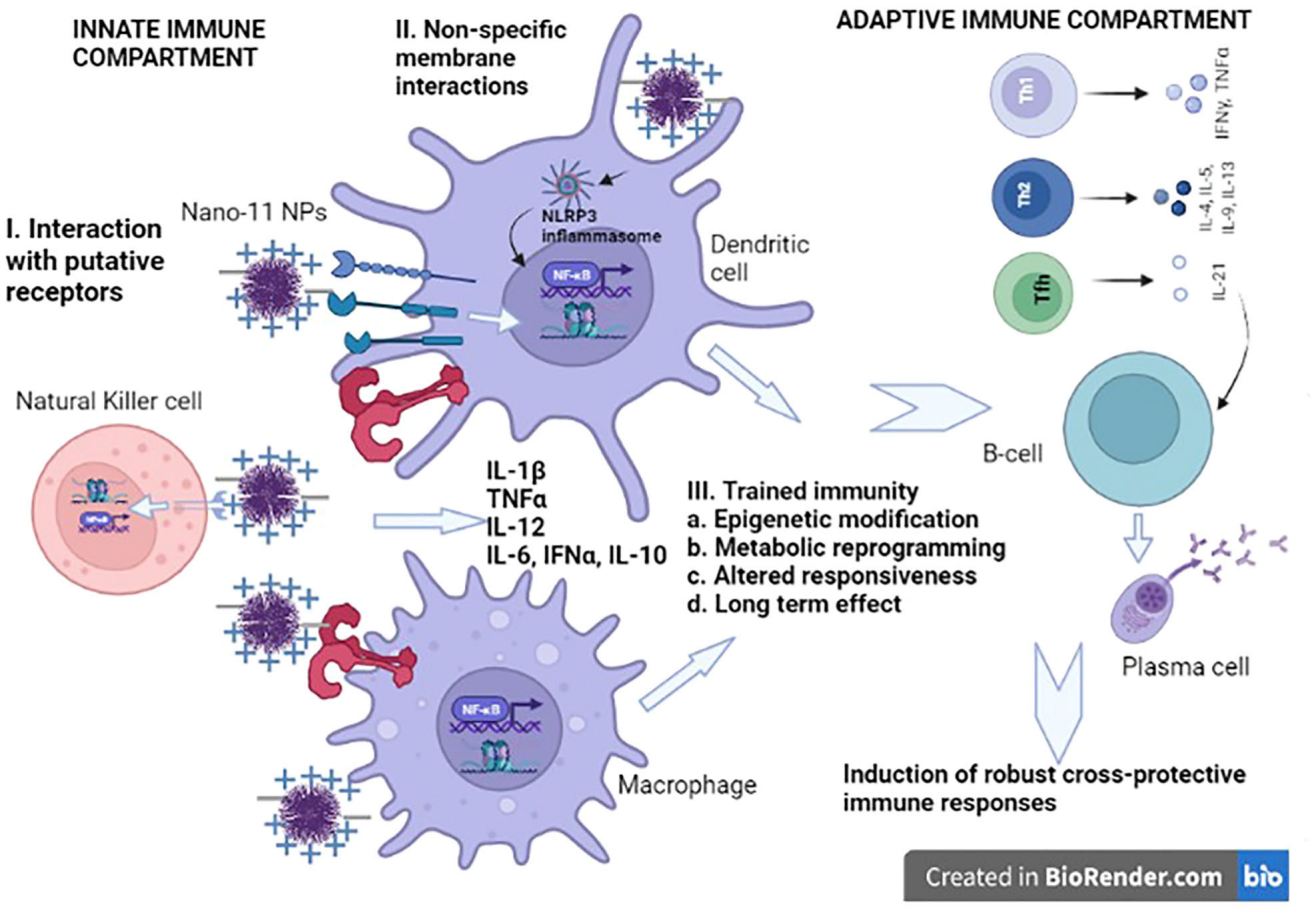
Summary of putative mechanisms of action of Nano-11 nanoparticles. Nano-11 NPs may exert their effects using three non-mutually exclusive modes. 1. Nano-11 could be recognized directly or indirectly by receptors such as NLRP3, dectin-1, dectin-2, DC-SIGN, complement receptor 3 (CR3), and TLRs. 2. Nano-11 may also mediate non-specific electrostatic membrane pertubations triggering intracellular signal transduction. 3. Nano-11 may elicit trained immunity via epigenetic modification, metabolic reprogramming, altered responsiveness leading to long lasting effects involving myeloid cells. These mechanism may lead to the induction of balanced Th1 and Th2 cytokines and induction of T follicular helper (Tfh) cells cells resulting in the elicitation of strong cross-protective immune responses. This model is based on both experimental evidence and hypotheses formulated from published literature.

Nano-11 slows down the diffusion of adsorbed antigens from the injection site after intramuscular injection of mice. This allows for the recruitment of antigen-presenting cells through the induction of local inflammation which take up the NPs and antigen and carry them to the draining lymph nodes (11). Interestingly, the kinetics of the recruitment of inflammatory cells following injection of Nano-11 showed the accumulation of primarily monocytes and macrophages with few neutrophils and nearly complete disappearance of inflammatory cells after 2-3 weeks (11). This contrasts with the inflammation induced by intramuscular injection of aluminum hydroxide adjuvant in which neutrophils are the most common cells during the first 24 hours followed by the accumulation of macrophages which can persist for months (10, 15). The transient inflammatory response is consistent with the lack of local reactogenicity and the excellent safety profile of Nano-11. Nano-11 directly activates antigen-presenting cells *in vitro* as indicated by increased expression of CD80, CD86 and major histocompatibility complex II (MHC II) on mouse and porcine dendritic cells (DCs) and human monocytic THP-1 cells (9). Nano-11 also induces the secretion of interleukin 1β (IL-1β) from lipopolysaccharide (LPS)-primed DCs and THP-1 cells through the activation of the NLR family pyrin domain containing three (NLRP3) inflammasome (4, 9). At this time, it is not clear whether the activation is mediated by interaction with specific receptors or non-specific binding of the cationic NPs to the negatively charged cell membranes resulting in destabilization that may trigger cell signaling (**Figure 1**).

## Nano-11 preparation and applications

**Figure 2:**
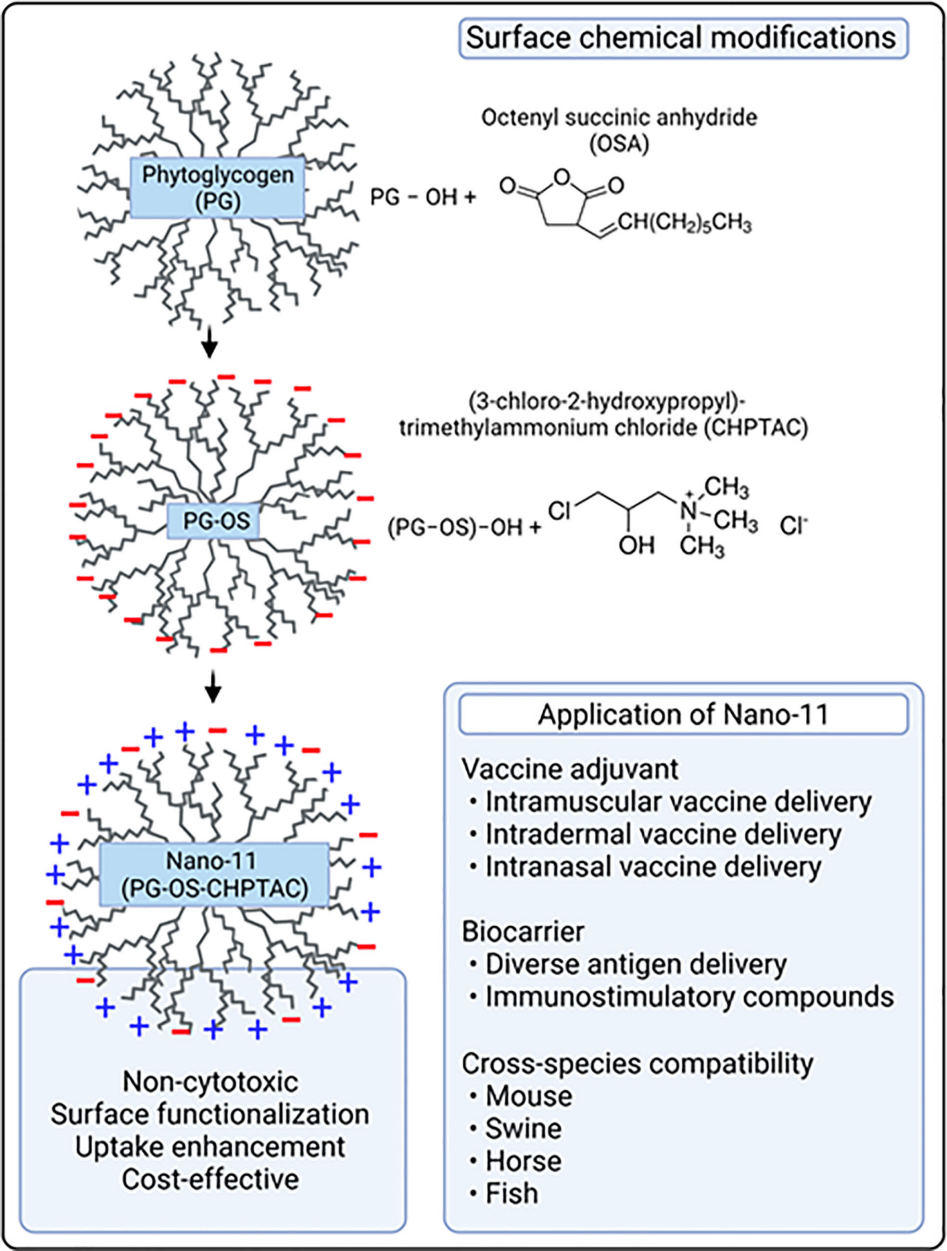
Summary of Nano-11 preparation and applications.

Nano-11 is prepared by two chemical modifications from PG. Treatment of PG with octenyl succinic anhydride provides a negative surface charge and hydrophobicity, whereas excess CHPTAC gives the final Nano-11 preparation an overall positive surface charge (**Figure 2**). Others have prepared cationic PG-derived nanoparticles by a single step treatment of PG with glycidyltrimethylammonium chloride, but these nanoparticles were reported to lack immunostimulatory activity (7). HH is coinventor of a US patent on Nano-11 (US2017/0128567).

## Nano-11 as vaccine adjuvant for intradermal immunization

Intradermal immunization is an attractive route of vaccine delivery as it targets tissue rich in antigen-presenting cells which may generate a more robust and qualitatively enhanced response compared with intramuscular or subcutaneous injection (16). In addition, intradermal immunization avoids the use of needles which accounts for significant healthcare costs due to accidental injuries, the need for adequate training and the cost of disposal (17). However, the lack of suitable vaccine adjuvants for intradermal delivery technologies has slowed the widespread use of intradermal vaccines. Nano-11 alone or combined with the stimulator of interferon genes (STING) agonist cyclic-di-AMP (cdAMP) induced a robust immune response following intradermal vaccination of mice and pigs (9). A significant dose sparing effect was observed with intradermal vaccination compared with intramuscular injection. While Nano-11 alone induces primarily a T-helper2 (Th2) response, the combination with cdAMP generated both T-helper1 (Th1) and T-helper17 (Th17) responses. The induction of a significant number of IFN-γ^+^ CD8 T-cells in response to immunization with ovalbumin and Nano-11/cdAMP suggests that the combination adjuvant promoted cross-presentation of protein antigens.

## Nano-11-adjuvanted vaccines against swine influenza

Based on the ability of Nano-11 to activate porcine monocyte-derived dendritic cells and to enhance the immune response to adsorbed ovalbumin following intramuscular and intradermal vaccination (9, 12), we assessed the efficacy of Nano-11 as an adjuvant for swine influenza virus (SIV) vaccines. SIV is a leading cause of morbidity and mortality in swine and a potential zoonotic risk as recombination of SIV with other influenza viruses can create new viruses of pandemic potential. SIVs circulating in swine herds continuously evolve and current commercial vaccines are not effective in protecting against virus types that are not included in the vaccines. Therefore, there is an urgent need for vaccines that induce cross-protection against heterologous strains. Intranasal (IN) administration of piglets with inactivated H1N2 SIV adsorbed on Nano-11 elicited significant numbers of Th1 and Th2 CD4 T-cells among peripheral blood mononuclear cells (PBMCs), and cross-reactive SIV-specific secretory IgA (SIgA) in nasal secretions (12). The frequencies of IFNγ^+^ CD4 memory T-cells and CD8 T-cells were significantly enhanced following challenge with heterologous H1N1. These changes were accompanied by a slight reduction of influenza symptoms, pneumonic lesions, and moderate decrease in respiratory tract H1N1 virus load.

In an effort to further enhance the efficacy of the SIV vaccine, we combined Nano-11 with the TLR3 agonist poly(I:C) which adsorbs readily to the positively charged α-D-glucan NPs (8). Intranasal immunization of piglets with inactivated H1N2 formulated with Nano-11/poly(I:C) elicited a significant amount of SIV cross-reactive SIgA antibodies in nasal secretions and lungs in contrast to a commercial multivalent SIV vaccine injected intramuscularly (8). In addition, piglets vaccinated intranasally with Nano-11/poly(I:C) had high titers of virus-neutralizing antibodies in the bronchoalveolar lavage fluid and increased expression of Th1 and Th2 cytokines in the tracheobronchial lymph nodes. Both the intranasal H1N2 SIV/Nano-11/poly(I:C) and intramuscular multivalent SIV vaccine partially reduced microscopic lung lesions and virus titers in the respiratory tract following challenge with H1N1 (8). These results show that the Nano-11/poly(I:C) combination adjuvant stimulated cross-reactive antibody and cytokine responses in the respiratory tract and support the further development of this adjuvant for intranasal SIV vaccines (8).

Currently available commercial intramuscular SIV vaccines not only fail to induce cross-protective immunity and mucosal SIgA required to reduce respiratory tract infection and virus shedding but are also susceptible to interference by maternally derived antibodies (MDA) in piglets born to vaccinated mothers. Intranasal vaccination of MDA-positive piglets with inactivated H1N2 SIV combined with Nano-11/poly(I:C) induced cross-reactive SIgA and IgG antibodies in the lungs. In addition, the H1N2 SIV vaccine combined with Nano-11/poly(I:C) elicited a robust antigen-specific polyfunctional, cross-protective cell-mediated immunity in the tracheobronchial lymph nodes (13). This is indicated by the increased number of SIV-specific IFN-γ^+^ and TNF^+^ CD8 T-cells and IL-17^+^ CD4 T-cells. These studies suggest that intranasal vaccination against SIV is less susceptible to interference by MDA.

## Discussion

Alpha (α)-D-glucans are linked together by α-glycosidic bonds aligned in the axial position. Whereas α-1,6 bonds confer flexible and relaxed structures, α-1,4 bonds impart helicoidal configuration. The immune cell receptors mediating their recognition, uptake, and processing are not well characterized. Structural properties such as molecular weight (MW), polymer length, degree of branching, type of glycosidic bonding, surface charge, purity, and solubility impart immunomodulation. The molecular weight of glycogen plays a significant role in the immune stimulation. Whereas the lower molecular weight glycogen (5000-6500 K) activates macrophage cell lines, their high molecular weight counterparts (greater than 10,000 K) fail to induce activation. Cytokines such as IFN-γ are important because they facilitate uptake of low MW glycogen and activation of macrophages (18, 19).

Carbohydrate-based NPs such as Nano-11 simulate the particulate nature of viral and bacterial antigens facilitating efficient recognition by the innate immune system. When a vaccine antigen is presented in a repetitive pattern on a nanoscale, it mimics the surface of the pathogens resulting in enhanced immune recognition, uptake, retention in the draining lymph nodes, and robust induction of antigen-specific responses. Furthermore, carbohydrates by virtue of their abundance in various cellular structures play a significant role in cell signaling, adhesion, movement, recognition, morphological transitions, and differentiation and hence contribute to their biocompatibility. Carbohydrate side chains and branches contribute to flexibility leading to enhanced uptake of antigens by antigen-presenting cells. Properties such as simple fabrication, scalability, mucoadhesive property, safety, natural abundance, solubility, availability of chemical moieties for functionalization, and particulate form make α-D-glucans attractive candidates for mucosal adjuvants (1, 20). Interestingly, our methodology of two-step chemical modification confers intrinsic mucosal adjuvanticity to Nano-11 nanoparticles and this is the uniqueness of our preparation. However, when Nano-11 nanoparticles are generated by other methodology as reported in two recent publications, there is no intrinsic mucosal adjuvanticity. The underlying mechanisms should be studied in detail.

Investigations of the immunomodulatory effects of microbial α-D-glucans [water-soluble and water-insoluble generated from *Streptococcus sobrinus* strain 6715 (21)] reveal that receptors such as dectin-1, dectin-2, complement receptor 3 (CR3), and Toll-like receptors (TLRs) could play critical roles in the recognition and uptake of α-glucans. Other studies have reported that dendritic cell receptor such as dendritic cell-specific intercellular adhesion molecule (ICAM)-grabbing non-integrin (DC-SIGN) also plays a significant role (18, 21–23). The activation of the innate immune system by these pattern recognition receptors (PRRs) induces and instructs the activation of the adaptive immune system.

Another possible mechanism that may contribute to the protection of vaccines containing α-D-glucans is the induction of trained immunity. Trained immunity or innate immune memory refers to the transiently heightened state of activation of the innate immune system leading to upregulation of secondary responsiveness to subsequent stimulations (identical or unrelated stimuli) in a relatively stable way. It is characterized by epigenetic and metabolic reprogramming of innate immune cells such as myeloid cells, monocytes, and innate lymphoid cells like natural killer (NK) cells and is systemically elicited at the level of bone marrow progenitors. It represents the adaptation of innate immune cells through epigenetic, metabolic, and functional reprogramming of transcriptional pathways. Trained immunity is usually reversible and lasts for a shorter duration than adaptive immune memory due to the limited life span of myeloid cells. However, the longer life span of trained macrophage and myeloid precursors in the bone marrow niche contribute to the longevity of trained immunity. Furthermore, trained immunity can be represented by augmented (training) or suppressed (tolerance) innate immune responses (24). Trained immunity-based vaccines offer several advantages such as broader non-specific protection against different pathogens based on trained innate immune cells and intrinsic adjuvant that elicit augmented adaptive immune responses to their own antigens and to the bystander antigens as well (25). Recently, trained immunity has been demonstrated in porcine monocytes *in vitro* (26). Recent studies have shown that cationic phytoglycogen nanoparticles can be used as a delivery vehicle for the TLR3 agonist poly(I:C) and induces activation of innate immune cells following oral delivery in rainbow trout (14). The peripheral triggering of innate immune memory in mouse alveolar macrophages following an adenoviral respiratory infection in the mucosal tissues has also been reported and this was facilitated by IFN-γ generated by CD8^+^ T-cells. This observation posits an adaptive-to-innate immune reverse signaling for the stimulation of trained immunity (27). Hence it is plausible that Nano-11 may elicit trained immunity to induce robust cross-protective adaptive immune responses to vaccine antigens. The mechanistic basis of the mode of action of different Nano-11-based vaccine formulations should be investigated in depth using systems vaccinology approaches. Such studies can identify the innate and adaptive molecular signatures to predict vaccine efficacy and gain mechanistic insights. These strategies aid in the design, development and testing of rational vaccines, and hence progress towards the realization of the full potential of Nano-11-based vaccine formulations.

## Author contributions

VP and JH-F wrote the article; HH and GR edited the article. All authors contributed to the article and approved the submitted version.

## Funding

This work was supported by United States Department of Agriculture, National Institute of Food and Agriculture (USDA–NIFA) Agriculture and Food Research Initiative Competitive Grant 2019-67015-29814 (HH and GR) and USDA–NIFA Hatch formula funds from Project IND020164H (HH).

## Conflict of interest

The authors declare that the research was conducted in the absence of any commercial or financial relationships that could be construed as a potential conflict of interest.

## Publisher’s note

All claims expressed in this article are solely those of the authors and do not necessarily represent those of their affiliated organizations, or those of the publisher, the editors and the reviewers. Any product that may be evaluated in this article, or claim that may be made by its manufacturer, is not guaranteed or endorsed by the publisher.
